# The airborne mycobiome and associations with mycotoxins and inflammatory markers in the Norwegian grain industry

**DOI:** 10.1038/s41598-021-88252-1

**Published:** 2021-04-30

**Authors:** Anne Straumfors, Sunil Mundra, Oda A. H. Foss, Steen K. Mollerup, Håvard Kauserud

**Affiliations:** 1 Department of Chemical and Biological Work Environment, National Institute of Occupational Health, P.O. Box 5330, 0304 Majorstuen, Oslo, Norway; 2 Department of Biosciences, Faculty of Mathematics and Natural Sciences, University of Oslo, Oslo, Norway; 3 Department of Biology, College of Science, United Arab Emirates University (UAEU), P.O. Box 15551, Al Ain, Abu Dhabi, UAE

**Keywords:** Air microbiology, Microbial communities, Fungi

## Abstract

Grain dust exposure is associated with respiratory symptoms among grain industry workers. However, the fungal assemblage that contribute to airborne grain dust has been poorly studied. We characterized the airborne fungal diversity at industrial grain- and animal feed mills, and identified differences in diversity, taxonomic compositions and community structural patterns between seasons and climatic zones. The fungal communities displayed strong variation between seasons and climatic zones, with 46% and 21% of OTUs shared between different seasons and climatic zones, respectively. The highest species richness was observed in the humid continental climate of the southeastern Norway, followed by the continental subarctic climate of the eastern inland with dryer, short summers and snowy winters, and the central coastal Norway with short growth season and lower temperature. The richness did not vary between seasons. The fungal diversity correlated with some specific mycotoxins in settled dust and with fibrinogen in the blood of exposed workers, but not with the personal exposure measurements of dust, glucans or spore counts. The study contributes to a better understanding of fungal exposures in the grain and animal feed industry. The differences in diversity suggest that the potential health effects of fungal inhalation may also be different.

## Introduction

Grain elevators and animal feed mill workers are exposed to grain dust and its heterogeneous contents, including fungal bioaerosols^1,2^. The inhalation of grain dust and its components may induce allergy and inflammation and impair lung function^3–9^, although an inconsistency in exposure–response relationships has been observed between studies. There may be several reasons for this variation; (1) the differential health effects of individual grain dust components; (2) differences between methodologies in grain dust composition studies^2,10,11^; (3) limited exposure characterization in epidemiological studies; (4) health effects that are too coarse/general to find associations with specific exposure measurements, and (5) healthy worker selection in occupational studies.

The role of microbial exposure diversity in inflammation and the development of chronic respiratory disease associated to organic dust exposure has been emphasized by several studies pointing towards the microbial composition as being decisive for health effect^12–17^. Hence, an improved characterization of the airborne microbial diversity is likely to improve the basis for understanding the cause-effect-relationship of occupational disease. The fungal exposure level in the Norwegian grain industry has been shown to exceed the proposed occupational exposure limit (OEL) for general fungal spores of 10^5^ spores m^−3^^18^, suggesting that fungal exposure may contribute to health effects. As different fungi may produce mycotoxins and other metabolites with different potency and induce various immunological responses, it is important to know the fungal species that are present, and not only the total spore exposure level. The fungal diversity of the dust will be dependent on the sources of the grain and growth conditions in the surrounding locality, and the exposure may vary with season, tasks, technical and organizational conditions at the work place^19–21^. *Cladosporium* spp., *Aspergillus* spp., *Penicillium* spp., *Wallemia sebi*, and *Fusarium* spp., are fungi often identified in association with grain and grain dust^22,23^. The ITS1 metabarcoding have shown that the fungal diversity in wheat dust differs with farming systems and cultivars^21^, and among wheat dust-exposed populations, such as grain handlers and cattle raisers^24^. This difference has also been shown to be associated with differences in the antibody responses between the two worker populations^25^. Other response markers of microbial exposure, such as increases serum amyloid A and C-reactive protein has been shown to be affected by the species composition in green houses^26^. We have previously shown differences in inflammatory markers and circulating miRNAs in blood of exposed workers compared to unexposed controls without finding any association with exposure measurements^27,28^. However, the complete assemblage of fungal species in the Norwegian grain elevators and animal feed mills has not been properly evaluated. Obtaining improved temporal and spatial fungal diversity data is therefore critical to improve the knowledge of occupational exposure–response relationships.

The primary objectives of this study were to characterize the airborne mycobiome in Norwegian grain elevators and animal feed mills using DNA metabarcoding and assess to what extent it differs between different seasons and climatically different districts. Secondary objectives were to assess possible relationships between fungal diversity and previously reported mycotoxins in settled dust^2^, personal exposure measurements of dust, glucans and spores^11^, as well as inflammatory markers in the blood of grain dust exposed workers^27^.

## Results

### Sequence data characteristics and overall taxonomic composition

The quality filtered dataset, after excluding all replicates, negatives control and mock community samples, contained 2416 operational taxonomic units (OTUs) (15,180,054 reads). The number of reads per sample ranged from 43,831 to 5,069,491 (average = 690,002) and per OTU ranged from 1 to 3,684,497 (average = 6283). Ascomycota, with 59.2% of the OTUs and 68.2% of the reads, was the most common phylum detected in the grain mill samples, followed by Basidiomycota (37.1% OTUs and 31.7% reads) and Zygomycota (0.8% OTUs and < 0.1% reads) (Fig. S1; Table S1). The phyla Glomeromycota, Chytridiomycota, and Rozellomycota were detected in very low proportions (all < 0.1% reads and OTUs). The most common orders (> 4% of total reads) detected in Ascomycota were Capnodiales (31.7%), Pleosporales (9.2%), Hypocreales (8.4%), Xylariales (6.5%) and Microascales (4.6%), whereas Tremellales (17.9%), Sporidiobolales (7.8%) and Cystofilobasidiales (3.2%) were most common in Basidiomycota. The most common OTU in grain mill air, an OTU with 100% identity towards *Cladosporium herbarum*, a widespread plant pathogen, contained 24.3% of all reads.

### Seasonal and climatic effect on fungal diversity patterns

**Figure 1:**
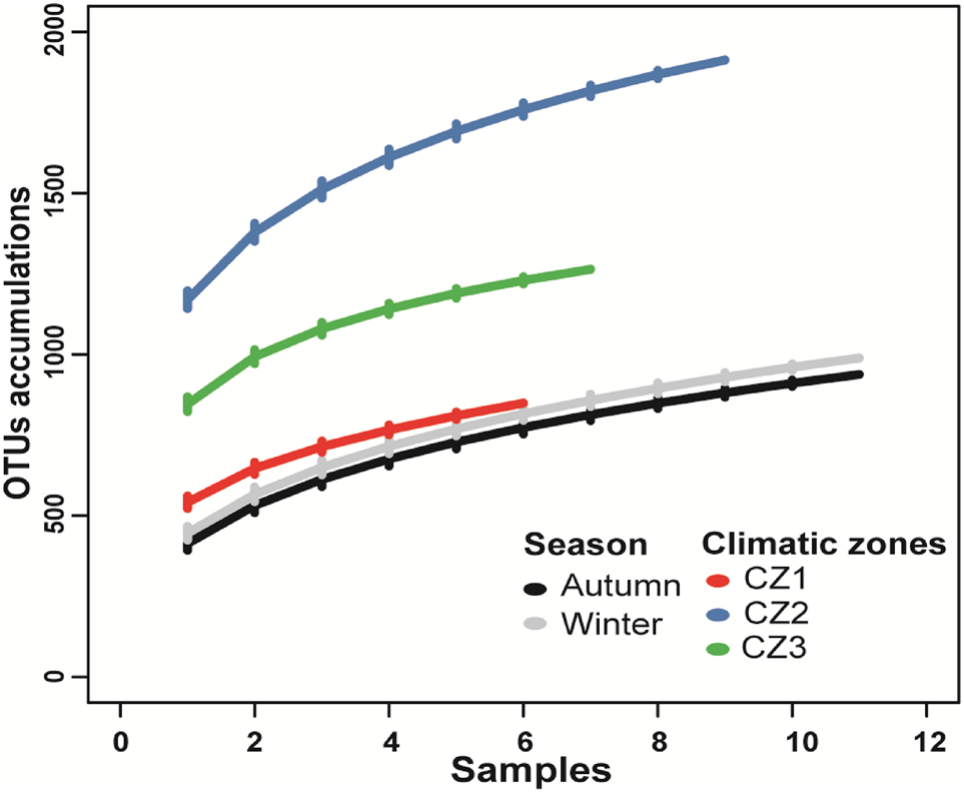
OTU accumulation curves for sequencing depth against OTU richness in different seasons (autumn and winter), and in three climatic zones, for the fungal composition recovered from industrial grain mill air.

The average OTU richness per sample was 239 ± 73 (average ± SD). The accumulation curve showed that the total number of OTUs detected from winter samples were relatively higher compared to autumn (Fig. 1), but the difference was non-significant in the ANOVA analysis (Table 1; Fig. S2). The sample with the highest species richness was from grain mills located in the continental subarctic climate of the eastern inland of Norway, with short summers and snowy winters (hereafter called climatic zone two or CZ2), and lowest in samples from grain mills located in the continental subarctic climate of central coastal Norway, with short growth season and lower temperature (hereafter called climatic zone one or CZ1) (Fig. 1). However, on average, significantly higher richness (ANOVA, *p* = 0.014) was observed in the samples from grain mills located in the humid continental climate of the southeastern Norway (hereafter called climatic zone three or CZ3) (281 ± 58) followed by CZ2 (251 ± 78) and CZ1 (171 ± 32) (Table 1; Fig. S3). In contrast, total abundances of the top 10 most common OTUs varied significantly between both seasons (being high during autumn) and climatic zone (being more abundant in zone one followed by two and three) (Table 1; Figs. S2, S3). Shannon diversity and evenness index values were significantly higher in winter compared to autumn, but differences among climatic zones were not significant for both indexes (Table 1; Figs. S2, S3).

**Table 1:** Effect of different seasons (autumn and winter) and three climatic zones on airborne fungal richness, Shannon diversity index, evenness and abundance of the 10 most common operational taxonomic OTUs (OTUs) in industrial grain mills.

Variables	Climatic zone		Season	
	F-value	*P* value	F-value	*P* value
Richness	5.40	**0.014**	1.30	0.268
Shannon	1.64	0.221	6.57	**0.019**
Evenness	0.65	0.529	4.82	**0.040**
Abundance of 10 OTUs	5.64	**0.012**	4.52	**0.046**

Statistically significant differences among studies factor variables were analyzed using ANOVA and Tukey’s HSD post-hoc test.

Bold values represent differences significant at a *P*-value < 0.05.

### Seasonal and climatic effect on fungal community structural patterns

**Figure 2:**
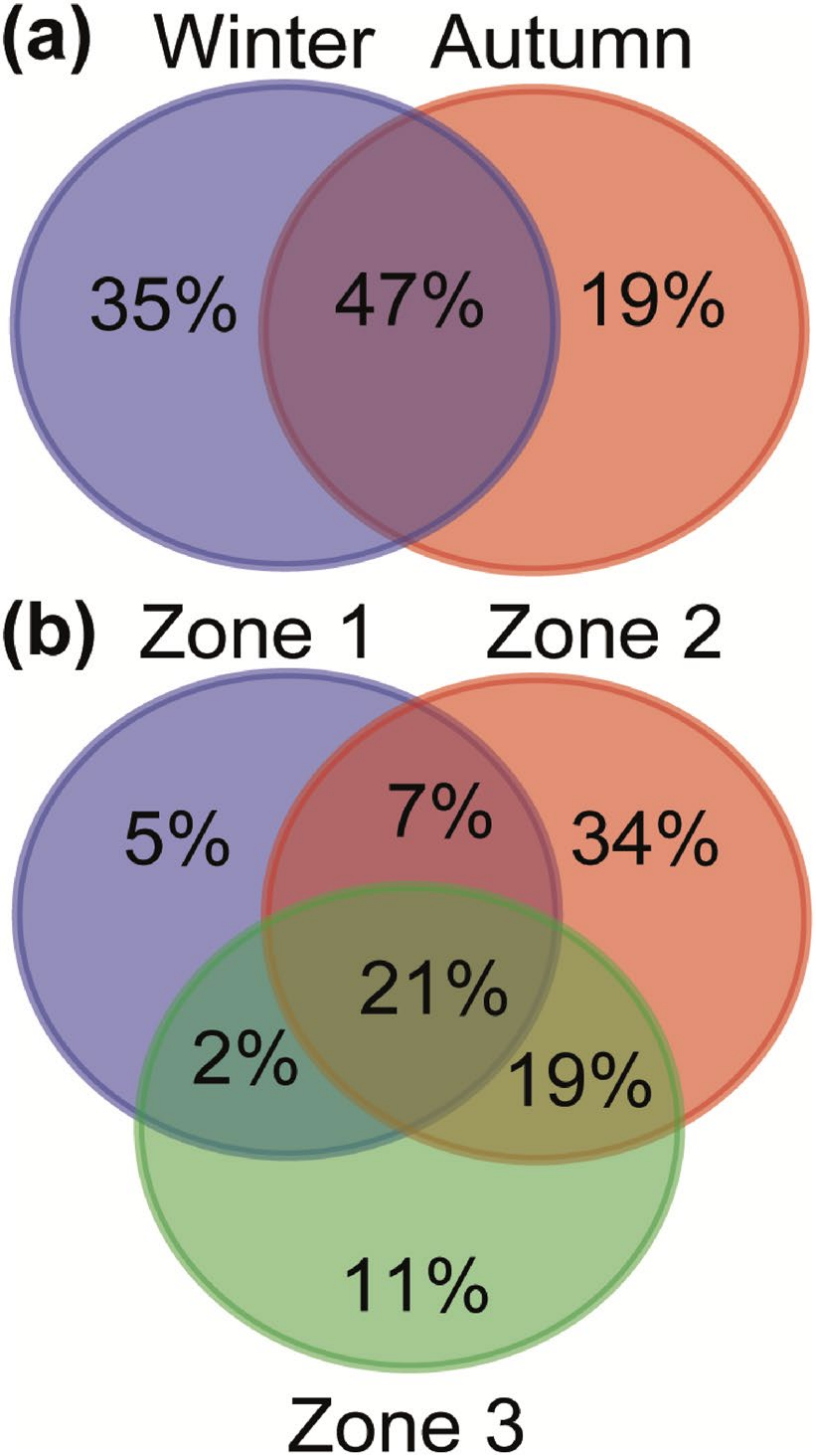
Percentage of shared and unique operational taxonomic units (OTUs) between different (**a**) seasons (autumn and winter) and (**b**) three climatic zones, for the fungal composition recovered from industrial grain mill air.

**Figure 3:**
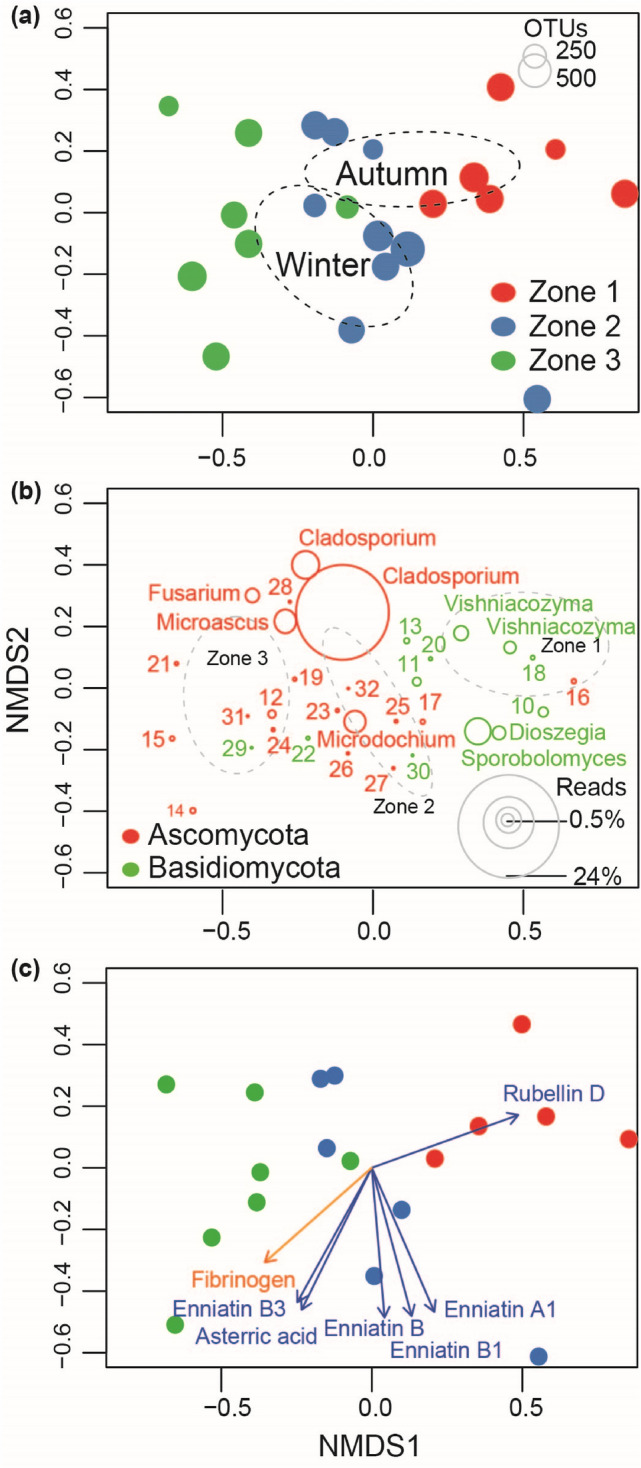
Nonmetric multidimensional scaling (NMDS) ordination analysis of airborne fungal communities in the Norwegian grain industry. (**a**) Plot represents air samples (n = 22) color coded by climatic zone (three) and ellipses representing the 95% confidence interval for the centroids of two different sampling seasons (autumn and winter). (**b**) Plot showing fungal operational taxonomic units (OTUs) composition of industrial grain mill air. The ordination plot is based on all fungal OTUs present, but only the most common 32 OTUs are shown here, which accounted for 86% of the total reads (m = million reads). (**c**) Plot represents industrial grain mill air samples (n = 18), from which mycotoxin data could be collected. Vectors (mycotoxin [blue] and fibrinogen [orange] variables) had significant effects (p < 0.05) on the ordination configuration. Numerical prefixes represent OTUs with taxonomic identity to following genera: *Itersonilia* (10), *Vishniacozyma* (11), *Gibberella* (12), *Vishniacozyma* (13), unclassified Phaeosphaeriaceae (14), *Alternaria* (15), *Aspergillus* (16), *Gibberella* (17), *Vishniacozyma* (18), unclassified Ascomycota (19), *Dioszegia* (20), *Epicoccum* (21), *Sporobolomyces* (22), *Bipolaris* (23), unclassified Pleosporales (24), *Botrytis* (25), *Alternaria* (26), *Sclerotinia* (27), *Aspergillus* (28), *Dioszegia* (29), *Filobasidium* (30), *Gibberella* (31) and *Neoascochyta* (32).

The variation partitioning analysis revealed that different climatic zones accounted for highest amount of the variation (23%) in fungal community composition, whereas season contributed 4% to the total variation (Fig. S4). Variations explained by season and climatic zones were not shared and 74% remained unexplained. In line with the above results, we also found that 35% of the OTUs were unique to winter season and 19% were unique to autumn, while 47% were shared between both seasons (Fig. 2a). Among the climatic zones, 21% OTUs were shared, while the highest proportion of unique OTUs were detected in zone two (34%) and lowest in zone one (4%). We also observed that sharing of OTUs between climatic zone one and three was lowest, whereas between zone two and three was highest (Fig. 2b). Multivariate PERMANOVA and NMDS analysis resulted in the very similar structural patterns, and confirmed strongest impact of climatic zone on fungal community (R^2^ = 0.35; *p* < 0.001; Table 2). Samples from different climatic zones were well separated from each other and those from the same climatic zone were grouped together in ordination space (Fig. 3a). Marginally significant seasonal effect on fungal compositional pattern was also detected (R^2^ = 0.06; *p* = 0.05), where the 95% confidence interval ellipses for autumn and winter were isolated, denoting distinct composition (Table 2; Fig. 3a).

**Table 2:** Relative importance of different seasons (autumn and winter) and three climatic zones on the airborne fungal community composition in industrial grain mills, as revealed from the PERMANOVA analysis.

Variables	*df*	F-value	R^2^	*P* value
Climatic zones	2	5.25	0.35	**< 0.001**
Season	1	1.82	0.06	**0.045**
Residuals	18		0.59	

Bold values represent differences significant at a *P*-value < 0.05.

### Seasonal and climatic effect on fungal composition

**Figure 4:**
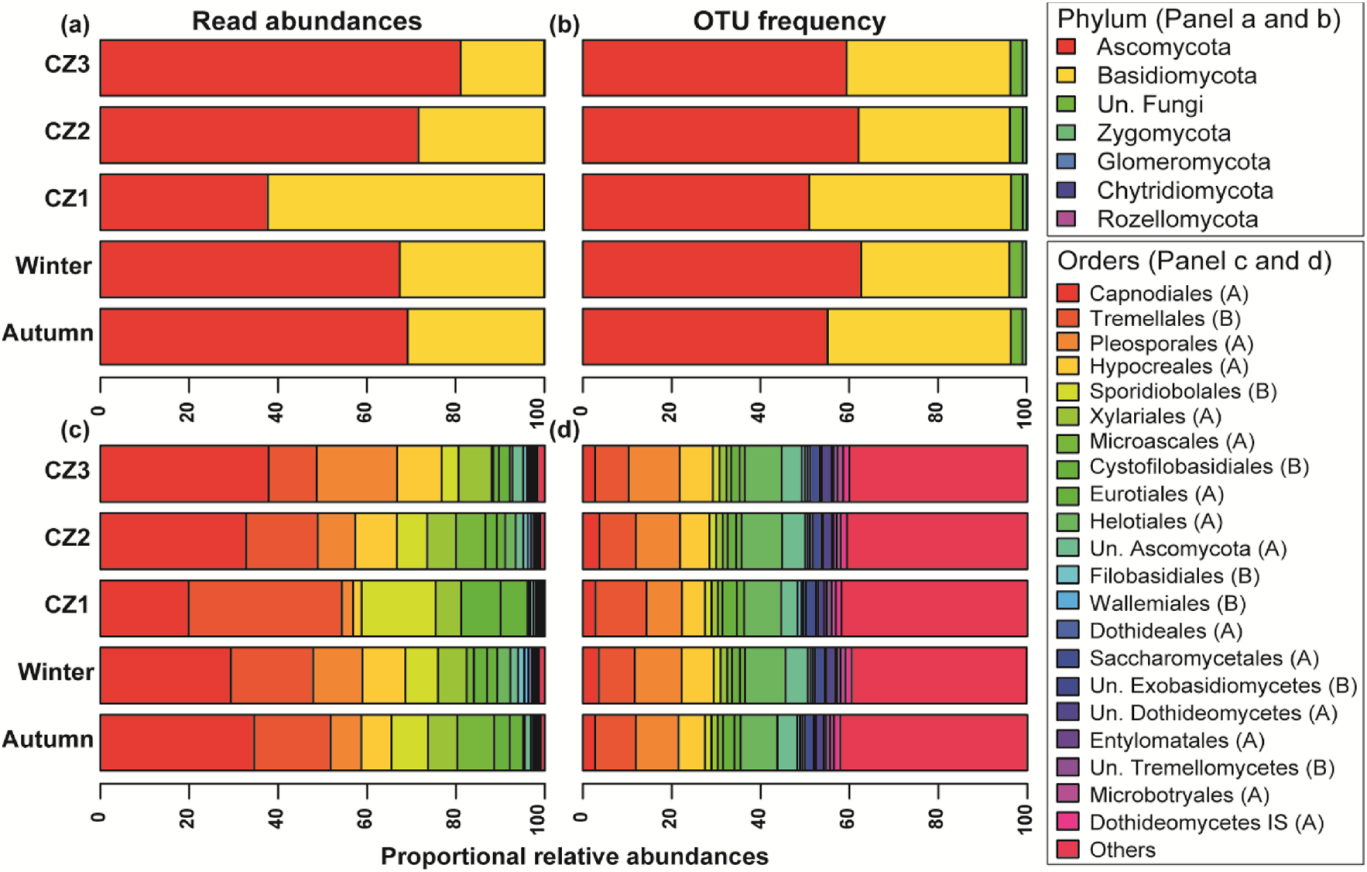
Proportional relative abundances (**a**,**c**) and OTUs frequency (**b**,**d**) of airborne fungal compositions at (**a**,**b**) phyla and (**c**,**d**) order level recovered from industrial grain mills. The data represent average reads per samples for individual variables. *Un*. Unidentified, *A* Ascomycota, *B* Basidiomycota.

**Figure 5:**
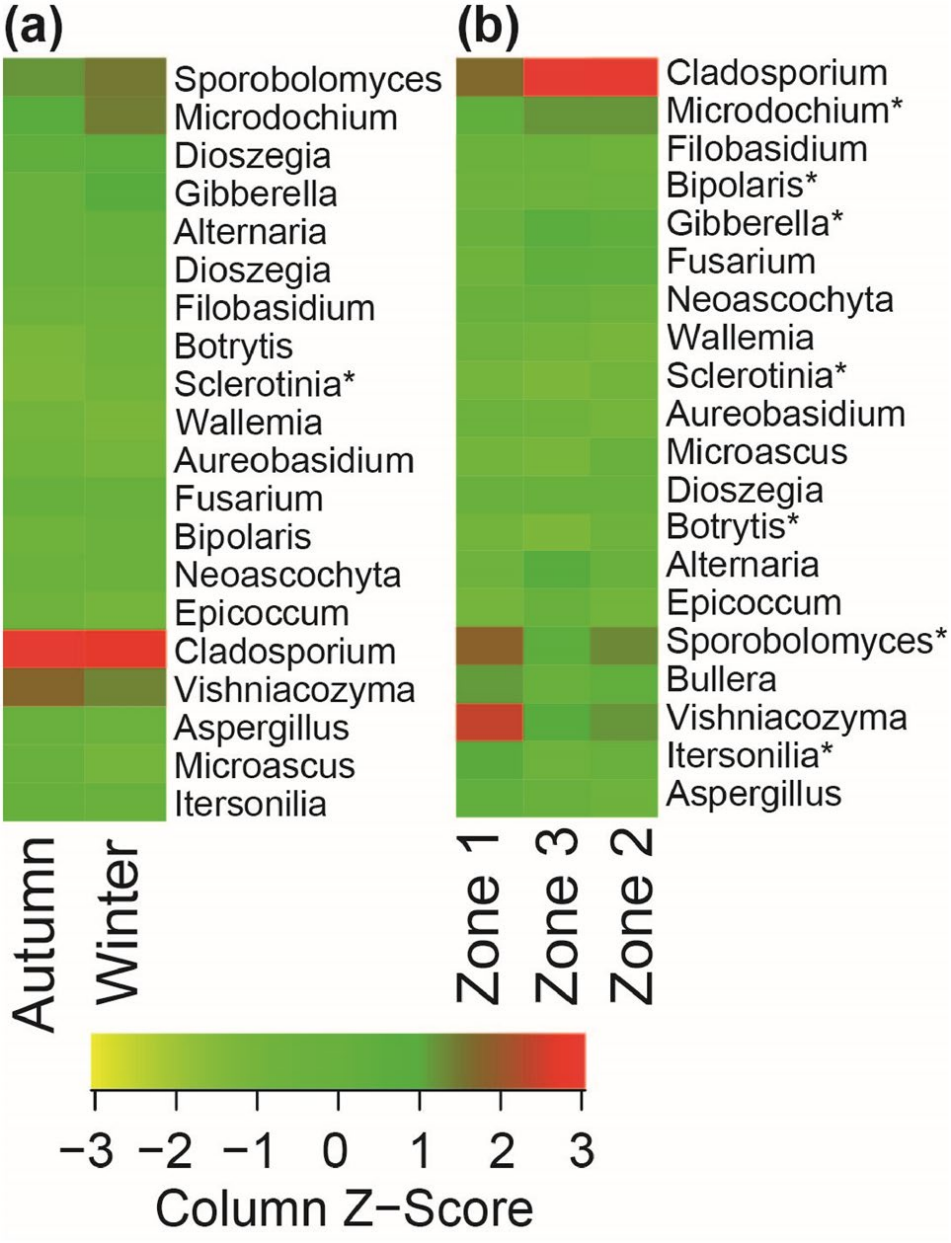
Hierarchical clustering-based heat plots for proportional abundances of different fungal genera that varied significantly between (**a**) seasons (autumn and winter) and (**b**) three different climatic zones in the air of Norwegian industrial grain mills. Genera with statistically significant differences (ANOVA and Tukey’s HSD post-hoc test) among studied factors are shown with “*”.

The shift in fungal composition at different taxonomic levels (from phylum to species level) also existed among both season and climatic zones. In autumn and winter seasons, the Ascomycota dominated in terms of read abundances (Fig. 4a; Table S1), but occurred in higher abundance during winter (62.7%), compared to autumn (55.1%) season (Fig. 4b; Table S1). The fungal composition at the order taxonomic level was highly season specific, with Capnodiales and Microascales (both ascomycetes) being most abundant during autumn whereas Pleosporales, Hypocreales Helotiales (all ascomycetes) and Tremellales (basidiomycetes) were more common in winter (Fig. 4c; Table S1). Order level composition in terms of occurrences was very similar between season (Fig. 4d; Table S1). Fungal genera also showed differential abundance distribution between seasons, where both *Bipolaris* and *Botrytis* were more abundant during winter compared to summer, and significant differences were recorded for *Sclerotinia* (F-value = 5.07; *p* = 0.035; Fig. 5a). The most common fungal OTUs also responded to the seasonal variations. In NMDS ordination space OTUs matched to species *Cladosporium herbarum* (OTU 1), *Cladosporium exasperatum* (OTU 9004), *Microascus* sp. (OTU 8) and *Fusarium* spp. (OTU 2) showed their species optima towards the samples from autumn whereas OTU 30 matching to species *Gibberella tricincta* was more abundant during winter (Table 3; Fig. 3b).

**Table 3:** Taxonomic affinity and occurrences of the 20 most abundant operational taxonomic units (OTUs) detected industrial grain mill air during different seasons (autumn and winter) and in three different climatic zones.

Samle_ID	Species	Order	Ide	Cov	Overall		Season (reads %)		Climatic zone (reads %)		
					Occurrences	Reads %	Autumn	Winter	CZ1	CZ2	CZ3
OTU_1	*Cladosporium herbarum*	Capnodiales (A)	100	100	17	24.3	25.5	23.2	15.9	25.0	28.6
OTU_9004	*Cladosporium exasperatum*	Capnodiales (A)	97	100	14	7.1	8.5	6.0	2.7	7.6	9.0
OTU_3	*Sporobolomyces ruberrimus*	Sporidiobolales (B)	100	100	21	6.7	7.8	5.9	16.3	5.7	2.7
OTU_5	*Microdochium nivale*	Xylariales (A)	98	100	20	5.6	5.6	5.6	5.4	5.6	5.6
OTU_8	*Microascus* sp.	Microascales (A)	89	100	7	4.3	8.0	1.3	0.0	6.2	0.1
OTU_9	*Vishniacozyma victoriae*	Tremellales (B)	100	100	18	3.7	3.9	3.6	7.4	3.3	2.5
OTU_2	*Fusarium sp.*	Hypocreales (A)	100	100	10	3.5	3.8	3.3	0.0	4.2	3.8
OTU_12	*Bullera crocea*	Tremellales (B)	100	100	20	3.4	3.3	3.5	8.2	2.9	1.3
OTU_331	*Vishniacozyma victoriae*	Tremellales (B)	99	100	19	3.1	3.5	2.7	8.0	2.5	1.0
OTU_14	*Itersonilia pannonica*	Cystofilobasidiales (B)	100	100	19	2.5	2.8	2.2	7.6	1.9	0.6
OTU_18	*Vishniacozyma* sp.	Tremellales (B)	100	100	19	2.1	1.9	2.4	3.4	1.9	1.9
OTU_25	*Gibberella tricincta*	Hypocreales (A)	100	100	18	2.0	1.5	2.3	0.8	1.8	3.5
OTU_580	*Vishniacozyma carnescens*	Tremellales (B)	98	97	18	1.4	1.3	1.5	1.3	1.5	0.8
OTU_26	*Phaeosphaeriaceae* sp.	Pleosporales (A)	100	95	13	1.4	1.3	1.5	0.5	1.0	3.8
OTU_41	*Alternaria metachromatica*	Pleosporales (A)	99	100	14	1.2	1.0	1.3	0.0	0.8	3.6
OTU_32	*Aspergillus appendiculatus*	Eurotiales (A)	100	100	7	1.2	1.3	1.0	4.0	0.8	0.1
OTU_30	*Gibberella tricincta*	Hypocreales (A)	100	100	12	1.1	0.3	1.9	0.6	1.5	0.2
OTU_6940	*Vishniacozyma* sp.	Tremellales (B)	97	100	17	1.1	1.1	1.0	2.8	0.9	0.3
OTU_34	*Ascomycota* sp.	Un. Ascomycota (A)	98	100	15	0.9	0.8	1.1	0.2	1.0	1.3
OTU_38	*Dioszegia fristingensis*	Tremellales (B)	98	100	19	0.9	0.8	1.0	1.0	0.9	0.9

For overall communities both occurrences and % total reads are shown, but for season and climatic zone only % total reads data is provided here. *Ide* % identification against reference sequence from UNITE database, *Cov* % sequence coverage.

Stationary air samples from companies in CZ2 and CZ3 were dominated by the fungal phyla Ascomycota both in terms of abundance and OTU frequency, whereas in CZ1 basidiomycetes were more common (Table 3; Fig. 4a,b). At an order level, fungal composition among climatic zones varied largely in terms of their abundances but not by their occurrence (Table 3; Fig. 4c,d). The Capnodiales, Pleosporales, and Hypocreales were more abundant in CZ3 followed by CZ2 and CZ1, whereas exactly the opposite pattern was observed for the Tremellales and Sporiodiobolales. Climatic zone also affected the distribution of different fungal genera (Fig. 5b). Abundances of *Microdochium* (F-value = 4.91; *p* = 0.019), *Gibberella* (F-value = 3.93; *p* = 0.037) and *Bipolaris* (F-value = 5.44; *p* = 0.013) were significantly higher in CZ2 and lower in CZ1; whereas *Sporobolomyces* (F-value = 3.84; *p* = 0.039) and *Botrytis* (F-value = 4.95; *p* = 0.018) were more common in CZ2 and less common in CZ3. Sequences placed in the genus *Itersonilia* were significantly more abundant in CZ1 as well as CZ2, whereas the pattern was opposite in CZ3 (F-value = 5.91; *p* = 0.037). OTUs belonging to *Sclerotinia* were only detected from CZ2 (F-value = 3.92; *p* = 0.037). Only one Ascomyceta OTU (identified as *Gibberella tricincta*) and the majority of the most common Basidiomyceta OTUs (matched to *Sporobolomyces ruberrimus, Vishniacozyma victoriae, Dioszegia crocea, Itersonilia pannonica* and *Cryptococcus* sp.) showed strong preferences in CZ1 and low preferences in CZ2 and CZ3. Abundance patterns was exactly opposite for most of the common Ascomyceta OTUs (matching to species *Cladosporium herbarum, Cladosporium exasperatum, Gibberella tricincta* and *Alternaria metachromatica*) restricted to CZ3.

### Correlation analyses between fungal compositional structure and occupational exposure and inflammatory markers

We investigated the relationship between fungal community structure and previously reported mycotoxins in settled dust (Table S2)^2^, as well as personal exposure to dust, fungal spores and 1,3-β-glucans (Table S3)^11^, and biomarkers of exposure in the blood of workers (Table S4)^27^. Using both procrustes (Correlation in a symmetric rotation = 0.37; *p* = 0.186) and mantel (r = 0.19; *p* = 0.137) analyses, we found that the overall mycotoxin level in grain dust was not significantly correlated with fungal community composition. However, individual mycotoxin analysis using envfit function with NMDS, showed that some mycotoxins were significantly correlated with the fungal community composition (Fig. 3c; Table S5). These mycotoxins were variants of enniatin (A1, B, B1 and B3), asterric acid, and rubellin D. Personal exposure levels of dust, fungal spores and 1,3-β-glucans were not significantly correlated with fungal composition (Table S5). Among all tested inflammatory markers in the workers blood (CC-16, SP-D, sP-selectin, IL-6, TNF-α, fibrinogen, sCD40L and CRP), only fibrinogen (R^2^ = 0.35; *p* = 0.04) was significantly correlated with the fungal compositional structure (Fig. 3c; Table S5).

## Discussion

In this study, the diversity and composition of fungal bioaerosols in stationary air samples derived from Norwegian grain elevators and animal feed mills was characterized. A broad spectrum of fungi was identified and the taxonomic groups characteristic of the grain industry in general belonged to the Ascomycota orders Capnodiales, Xylariales, Hypocreales, Pleosporeales and Eurotiales, as well as the Basidiomycetes orders Sporidiobolales, Tremellales, and Cystofilobasidiales. The airborne mycobiome identified in this study differed in fungal diversity and composition depending on season and geographically and climatically different zones. The fungal composition was related to some mycotoxins in certain zones, and to fibrinogen from the blood of exposed workers, but was not correlated with personal exposure measurements of dust, glucans or spore counts.

**Figure 6:**
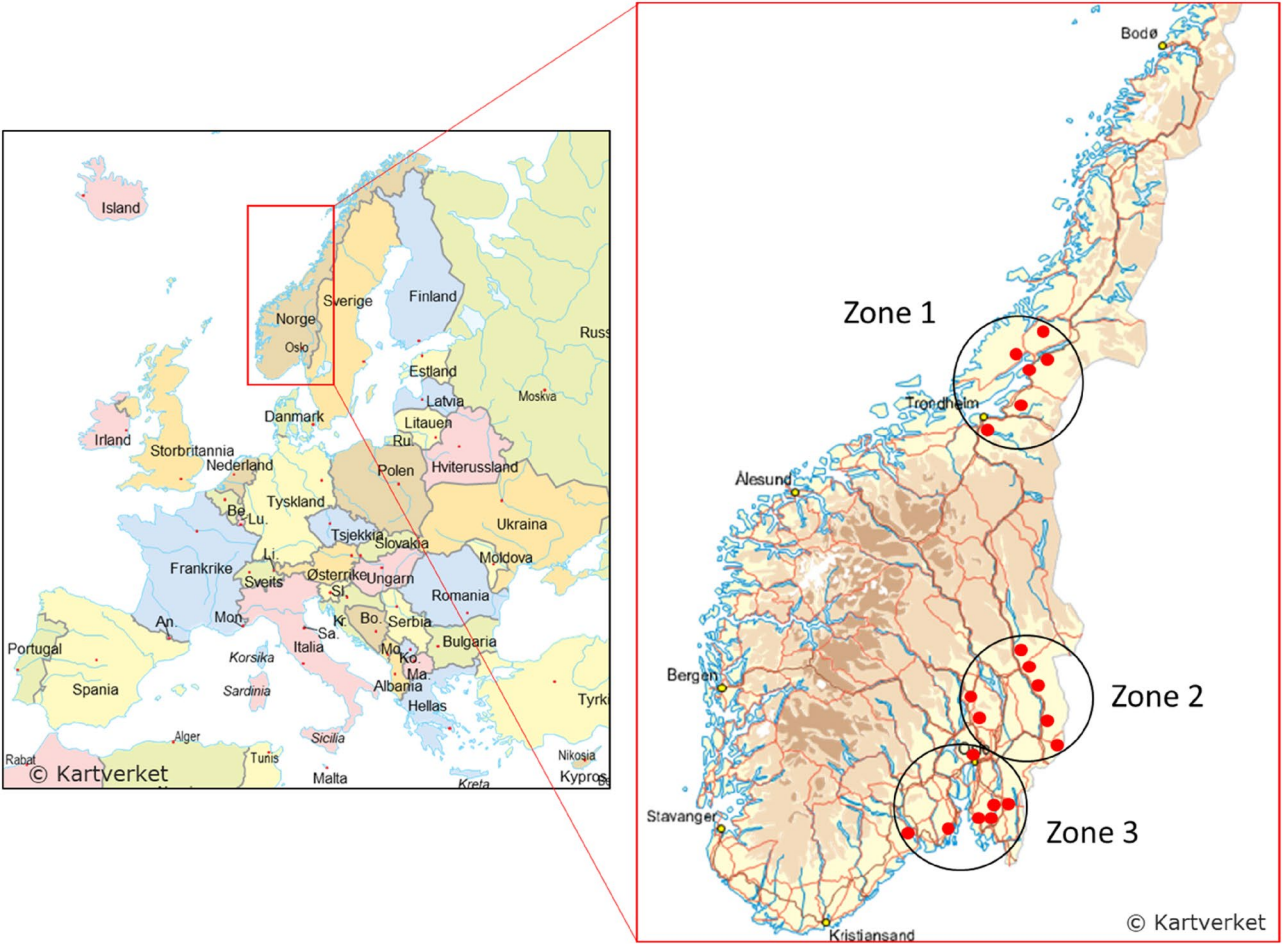
Map of the sampling locations. The sampling site spun over three geographically and climatically different districts; zone 1: the central coastal Norway (Trøndelag), zone 2: eastern inland of Norway (Viken, Innlandet), and zone 3: southeast Norway (Oslo, Viken, Vestfold and Telemark). All districts are located north of 59 degrees northern latitude. The figure consists of sections of illustration maps of Europe and Norway, respectively, provided under Public License by Geonorge Version 12.5.557 from https://kartverket.no/en/on-land/kart/illustrasjonskart, modified with the inclusion of red, filled circles representing each of the 20 companies included in the study and black circles representing each of the different zones with the belonging zone number.

Differences related to climatic zones accounted for most of the variation in airborne fungal community structure, explaining 23% of the variance, and shown by the distinct fungal community structure and composition of each of the zones. The zones represent differences in geographic location and climatic conditions that likely influence the occurrence and fungal growth in the surrounding environment, and subsequently in the locally produced grain that enters the grain elevators, of which the airborne dust is generated. Relatively homogenous compositions of fungal communities were found in wheat grain dust from 96 fields across 560 km^2^ in a Swiss study^21^. This shows that the geographic distance may not necessary be the strongest determinant for fungal diversity differences in general. However, an important role of geographical distances in structuring the fungal communities of other crop types, such as wineyards, have been indicated^29^. Whereas Pellissier and colleagues analysed the mycobiome of wheat dust, the present study included dust from wheat, rhy, oat and barley of various cultivars, which are likely to influence the diversity pattern differently^21,30^. Although all sampling zones are located north of 59 degrees latitude, and therefore have harsh winters, the climate is mildest in CZ3; the Southeast of Norway, followed by CZ2; Eastern inland of Norway and CZ1; the central coastal Norway (Fig. 6). It is therefore plausible that the airborne grain dust mycobiome of grain mills of the Southeast of Norway had higher fungal diversity in terms of species richness and Shannon diversity index than those of the Eastern inland of Norway or the central coastal Norway. CZ2 and CZ3 are geographically closer to each other and shared 19% of OTUs, whereas CZ1 shared only 7% OTUs with CZ2 and 2% with CZ3. CZ1 was also dominated by sequences placed in the phylum Basidiomycota, compared to the other districts that were dominated by the Ascomycota. This is also in contrast to Ascomycota species that are considered more robust, as CZ1 is considered a harsher environment in the northernmost district in the study. However, although short grain growth period, the coastal climate around the Trondheim fjord characterized by a mild climate, and prime agricultural district, the CZ1 may give better growth conditions for fungi placed in the Basidiomycota.

The distinct fungal compositional profiles between each climatic zone indicates that employees in different zones could be differently exposed. Fungal exposure assessments representative of all zones should therefore include measurements among the 21% of OTUs shared among the zones. However, it will be important to investigate what the fungal profile difference may imply for occupational health of the workers in the different zones.

We observed higher fungal diversity and evenness index values, and a tendency to higher richness, in winter compared to autumn. This result may be somewhat surprising, considering that autumn may represent more favorable growth conditions and higher rate of spore dispersal due to higher temperature and nutrient sources. It is also in contrast to other studies that have shown seasonal differences in fungal communities associated with sawmills^31^, plant leaves^32^, roots^33^ and soil^34^. However, the grain dust generated in winter results from handling more heterogeneous grain types received during winter than grain types that are received during autumn. As the grain at this time of year has already dried, and therefore will not have the need to go through drying processes at the elevators, the grain turnover at the mills may be higher, leading to more heterogeneous fungal dust. The grain also likely originates from several diverse Norwegian farms that has dried the grain themselves. Further, as the need for grain is larger than the local production, a considerable amount of grain imported from abroad is additionally received and handled, generating dust containing fungi from broad variety of geographic environments. Grain handlers would thus be exposed to different fungal species in autumn and winter, although the seasonal difference in species composition accounted only 4% of the variation.

A greater relative abundance of sequences placed in the phylum Ascomycota than Basidiomycota was detected in winter and autumn samples, which is in line with the view of Ascomycota species are more tolerant to environmental stress. In this case, this would not mean the harsh winter conditions, as previously indicated for the mycobiome in Norwegian sawmills^31^, but more likely the production process-related strain that fungi are exposed to during drying and moving the grain at the elevators and feed mills. However, we observed seasonal differences at the order and genus levels that may be explained by different nutrient preferences or tolerances of the identified fungi. The abundance of the 10 most common OTUs were higher in autumn than in winter. This relationship might be related to the evenness of the samples, i.e. if there are fewer species during winter, these will appear as more abundant relatively. Hence, the winter season at grain elevators and animal feed mills most likely represent the most diverse fungal airborne exposure potential.

The stability of mycotoxins make them remain in the dust although their fungal producers die and disintegrate. Species not producing mycotoxins may also be present. This can result in low correlations observed between mycotoxins and potential producers detected in the dust. Despite this, we observed a significant correlation between the fungal community structure and enniatins, asterric acid and rubellin D. Enniatins were present in high concentrations in the dust, whereas asterric acid and rubellin D were present in lower levels (Table S3). Rubellin D is a phytotoxin produced by *Mycosphaerella rubella*^35^ and *Ramularia collo-cygni*^36^, and has also antimicrobial, antiproliferal and cytotoxic properties. These fungi belong to the Capnodiales, the most abundant order of the study, and *Mycosphaerella* sp. occurred in 45% of the samples whereas *Ramularia* were found in 82% of the samples, thereof 64% *Ramularia collo-cygni* (32,508 total reads and 1478 average reads). Enniatins are depsipeptides that are naturally produced by the filamentous fungi in the genera *Fusarium*^37^, *Halosarpheia* and *Verticillium*^38^*.* The main producer, *Fusarium*, occurred in 91% of the samples (560,726 total reads and 6372 average reads), *Verticillium* only in 9% of the samples (n = 2; 42 total reads), whereas no *Halosarpheia* were observed. Enniatins have a range of biological activities, including anti-insectian, antifungal, antibiotic, and cytotoxic^39^. Asterric acid is an inhibitor of vascular endothelial growth factor (VEGF)^40,41^, and is produced by *Aspergillus* and *Penicillium* species, which were found in 82% and 55% of the samples, respectively.

Although the mean fungal spore exposure and 40% of the samples were above the proposed occupational exposure limit for fungal spores of 10^5^ spores m^−3^ (Table S4), the fungal species composition did not correlate with the spore counts, neither 1, 3-β-glucans nor dust exposure. As PCR-amplified and MiSeq-sequenced fungi will include hypha and fragments in addition to spores, a correlation with SEM counts of spores was neither expected. It is worth noting that results from metabarcoding represent a completely different picture than the traditional occupational exposure measurements, and the details obtained should be taken into consideration for refining the traditional methods into more targeted measurements. The cause-and-effect relationship between complex microbial occupational exposures and health effects is not clear, but the characterization of the microbial complexity of occupational exposures is a step on the way. The investigation of the relationship between the exposure diversity and inflammatory and anti-inflammatory exposure responses and potential health effects of complex exposure is an important next step towards increased understanding. Barrera and colleagues did indeed find differences in the exposure responses between grain handlers and cattle raisers that seemed to be related to differences in the airborne mycobiome^25^.

Several predominant fungal OTUs identified in this study belong to the genera *Aspergillus*, *Penicillium*, *Alternaria*, *Cladosporium* and *Wallemia,* which include plant and animal pathogenic and allergenic species that may cause respiratory effects^42–44^. Other common crop-plant associated and plant pathogenic fungi, such as species of the genus *Fusarium,* can have large impact on human health through their immunosuppressive, toxigenic, teratogenic, and carcinogenic mycotoxins^45^. OTUs from the Basidiomycota genus group *Cryptococcus* was found in all samples, but only 9% matched to the animal-pathogenic infectious species, *Cryptococcus neoformans*, that can cause cryptococcosis, a systemic fungal disease that affects internal organs and skin of immune deficient individuals^46^. The species *Vishniacozyma victoriae*, which has been suggested to be inversely associated with asthma^47^, was detected in the majority of the samples.

Although blood concentrations of both CC-16, IL-6 and fibrinogen were significantly different between exposed and controls (Table S5)^27^, none of the markers were associated with dust exposure or any other exposure component (fungal spores, endotoxins or 1,3-β-glucans) measured during work^27^. This was in spite of exposures to elevated bioaerosol concentrations^11^. However, when compared with the fungal species composition characterized in the present study, the fibrinogen concentration was associated with fungal diversity at company level. Apart from playing a role in the acute phase response to systemic inflammation, fibrinogen has been shown to be hydrolyzed by proteinases from *Aspergillus melleus* and other allergenic organisms to yield cleavage products that drive distinct antifungal mechanisms and initiates allergy (airway hypersensitivity, innate allergic inflammation and antifungal immunity) through Toll-like receptor 4 and CD11c^48^. Indeed, exposed workers had lower levels of fibrinogen compared with controls^27^, but although this may be a possible ongoing mechanism in the exposed workers, most employees in both study groups were within the reference area for fibrinogen of 1.5–4 mg mL^−1^. The restriction of the larger individual dataset of the inflammatory markers to the pooled dataset at company level represents a necessary limitation for the NMDS/PERMANOVA analyses. The results as such cannot be used to identify risk factors or quantify their significance. This has to be taken into consideration when interpreting the results. Further analyses beyond this point, such as the identification of specific fungal species or species profiles that represent a particular exposure–response should be conducted with quantitative epidemiological methods in the future.

In conclusion, this DNA metabarcoding study of the fungal ITS2 region in airborne grain dust provides insight into the complete spectrum of fungal bioaerosols across seasons and climatic zones in the Norwegian grain industry. This contributes to a better understanding of fungal exposures in the occupational workforce of the grain and animal feed industry. The differences in diversity suggest that the potential health effects of fungal inhalation may also be different. Fungal diversity differences should therefore be included in future risk assessments. This inclusion can lead to the development of improved exposure assessment methodologies to clarify exposure–response relationships.

## Materials and methods

### Sampling and sampling sites

Sixty-eight grain dust exposed workers at twenty companies of industrial grain elevators and compound feed mills participated in this study through the winter and autumn season of 2008. Four sets of samples were collected (Table 4): (1) Personal samples of the workers exposure to inhalable dust and bioaerosol components during a full work shift. The workers carried the sampling equipment during work, and the collected dust represented the workers inhalable exposure during all tasks performed that day; (2) Stationary full-shift sampling of airborne dust subjected to mycobiome analyses. Samplers were placed 1.5 m above ground at a fixed position assumed to be representative for the activity at each company; (3) Freshly settled grain dust from the production facilities subjected to mycotoxin analyses; (4) Blood samples from exposed workers. Blood were collected after work from the same persons that carried the equipment for exposure measurements.

**Table 4:** Overview of the acquisition and number of samples for each sample set.

Analyses	Sample material	Sampling equipment	Autumn			Winter			Total
			CZ1	CZ2	CZ3	CZ1	CZ2	CZ3	
Mycobiome	Full shift stationary aerosol samples	NIOSH one-stage cyclones	6	2	3	0	4	9	25
Bioaerosols^a, b^	Full shift personal inhalable aerosol samples	PAS-6 inhalable samplers	18	9	16	0	17	8	68
Mycotoxins	Newly settled dust	Spoon, brush and collection tubes	10	8	7	9	4	5	33
Biomarkers^a^	Blood sampled after work	Syringes and blood sampling tubes	18	7	11	0	19	13	68

^a^For bioaerosols and biomarkers, the number of samples equals the number of exposed workers.

^b^The number of bioaerosol samples represent parallel sampling for analyses of dust, fungal spores and β1 → 3-glucans. *CZ* climatic zone. The number of companies grouped in CZ1, CZ 2 and CZ3 was 6, 7 and 7, respectively. All samples were collected at the same day during winter and autumn 2008.

The sampling spun over autumn season and winter season, and three geographically and climatically different grain districts; (1) central coastal Norway, characterized by continental subarctic climate with short growth season, rain, low temperature and 850 day-degrees, (2) eastern inland of Norway, also with a continental subarctic climate, but with less rain, but more snow and about 1000 day-degrees, and (3) south-eastern Norway, with humid continental climate with humid and warm summers, and 1200 day-degrees (Fig. 6). The districts are referred to as climatic zone 1, 2 and 3, respectively, in the text. All districts are located north of 59 degrees northern latitude. The industrial process of the study plants has been described previously^19^. In grain elevators, various grain was loaded into the elevator, sorted, winnowed, dried, rotated, moved, stored and unloaded on a continuous basis. Grain moving was either air-driven (suck-and-blow), elevator-driven, or done by passive emptying by gravity. In compound feed mills, the grain was milled and mixed with other nutrients such as maize, calcium carbonate, fat, vitamins, and amino acids, pressed into feed pellets and filled into sacks or tanks. In autumn, a large part of the grain loaded into the grain elevator came from local producers and could include humid batches that needed drying. In winter season, the delivered grain had been dried on the farms before delivery, and the amount of imported grain was higher than in autumn.

### Stationary sampling of airborne dust for mycobiome analyses

Stationary sampling of airborne dust (n = 25 samples in total) was performed in 20 grain elevators and compound feed mills in Norway using NIOSH 1 stage samplers (NIOSH, Morgantown, USA) connected to SKC high flow portable battery-powered pumps (SKC Inc., Valley View, PA, USA) with an airflow of 4 L min^−1^. The samplers included a 1.5 mL Eppendorf tubes, collecting particles above 1.5 µm in aerodynamic diameter (theoretical particle size cut-off), and a 25 mm polycarbonate filter with pore size 0.8 µm placed in a 25 mm black IOM cassette (SKC Inc.), as a back filter, collecting particles smaller than 1.5 µm. The time of sampling was 235 to 475 min (mean 368 min). The samplers were placed in 1–2 m height at work sites representative for the activity of the companies.

### DNA extraction, amplification and sequencing

DNA was extracted from dust collected in the Eppendorf tube part of the sampling equipment. 150 µL cetyl trimethylammonium bromide (CTAB) buffer were initially added, and the DNA was extracted as previously reported^49^. Fungal ITS2 rRNA gene region was amplified using the fITS7a and ITS4^50,51^ primer combination. We followed similar PCR reactions setup and conditions as well as amplicon library preparation method as mentioned previously^49^. In addition to the 24 samples, individual cultured species, 4 parallels of a mock community, 2 field blanks, and a PCR negative control were also included in the sequencing run as control samples, and were used while curating the final OTU tables as shown before^31^. The mock community comprised fungal cultures of *Rhizopus microsporous*, *Cladosporium allicinum*, *Penicillium variotii*, *Fusarium graminearum*, *Aspergillus fumigatus* and *Wallemia sebi,* with known genome size, and was used to estimate index jumping in the sequencing run. Molecular data were generated using Illumina Miseq Paired-End (PE: 2 × 300 bp) sequencing.

### Mycotoxin analyses

Multiple mycotoxins and other metabolites of fungi were extracted from freshly settled grain dust (1.5–15 g) collected from the 20 grain elevators and animal feed mills, and analysed with LC–MS/MS. The analyses and results have been described previously^2^, and in the present study, the arithmetic mean concentrations from each company (Table S3) was used for correlations analyses with fungal compositional data.

### Aerosol exposure measurements and analyses

Fifty-six full-shift personal inhalable samples were collected with PAS-6 personal inhalable samplers^52^ and portable pumps (PS101; National Institute of Occupational Health, Oslo, Norway) for 6–8 h with a flow rate of 2 L/min. Samples were analyzed for grain dust, fungal spores and β-1,3-glucans as previously described^11^. In brief, dust was weighed, fungal spores were counted by scanning electron microscopy, and β-1,3-glucans were analysed by enzyme immunoassay. The results of the personal exposure have been published previously^11^. In the present study, the mean concentration of each aerosol component from each company (Table S4) was used for correlation analyses with fungal compositional data.

### Blood sampling and analyses of biomarkers

Blood samples were collected after work between 1 and 3PM. Club Cell protein 16 (CC-16), surfactant protein D (SP-D), SP-A, interleukin 6 (IL-6), serum P-selectin (sP-selectin), tumor necrosis factor-alpha (TNF-α), fibrinogen, C-reactive protein (CRP) and serum CD40 ligand (sCD40L) were analyzed by ELISA and reported as previously described^27^. In the present study, the mean concentration of each biomarker measured in the blood of workers at each company (Table S5) was used for correlation analyses with fungal compositional data.

### Bioinformatics processing

The Miseq sequence data were processed through the same bioinformatics pipeline as previously reported^49^. Taxonomic assignment were made by comparing the representative sequence against curated reference database UNITE v6^53^. The data treatment strategy generated an OTU table where all the control samples (extraction, negative and field blanks) were perfectly clean with no reads. Furthermore, mock composition represented by mock communities was as per our expectations. This indicated the appropriateness of the data treatment procedure to account for index hopping. Of total 25 samples, one sample failed during sequencing run, leaving 24 samples in the final dataset.

The dataset revealed non-normal spreading of reads among the samples (Fig. S5a) and distribution patterns of reads per OTU were also skewed, with only a few very abundant OTUs and many with very low abundances (Fig. S5b). Per sample rarefaction curves of OTU richness indicated that complete diversity was not captured for majority of the samples (Fig. S6). Additionally, a positive relationship was also observed between OTU richness and sequencing depth (R^2^ = 0.34; *p* = 0.004). To overcome this possible sequencing depth bias, we decided to normalize (43,837 reads per sample) the dataset for richness and diversity analysis, and were subsampled randomly. To account for inconsistencies in abundance measures and further to improve variance homogeneity dataset was arcsine-transformed prior to analyses. Two samples that had extremely low reads (35 and 2314 reads respectively) were removed from both community and diversity analysis, and 22 samples remained in final dataset, used for overall community analysis. Due to lack of mycotoxin data from four samples, only 18 samples were used while analyzing fungal and mycotoxin dataset. To achieve homogeneity of variances the values of the studied variables (mycotoxins in settled dust, airborne exposure levels of dust, fungal spores, and 1,3-beta glucan, and blood concentrations of CC-16, SP-D, SP-A, sP-selectin, IL-6, TNF-α, Fibrinogen, sCD40L and CRP) were transformed to zero skewness and expressed on a 0–1 scale prior to analyses.

### Statistical analysis

Unless stated otherwise, statistical analyses were performed in R v3.5.0^54^. Richness, Shannon and evenness diversity measures and total abundance the most common top 10 OTUs were calculated using R package *vegan*, and their differences between two seasons (autumn and winter) and three climatic zones were examined using ANOVA followed by a Tukey’s HSD post-hoc test from R package *agricolae* and visualized using violins plots. Seasonal and climatic zone differences in proportional abundances of different genera were tested using ANOVA (Benjamini–Hochberg FDR correction was applied) followed by a Tukey’s HSD post-hoc test and illustrated by heat plots based on hierarchical clustering using R package *vegan*. Species accumulation curves for number of accumulative OTUs for different seasons and climatic zones were calculated using function specaccum from R package *vegan* with 10,000 permutations. Overall taxonomic composition of airborne fungi (up to family level) was visualized with heat tree generated using R package Metacoder^55^.

The Bray–Curtis dissimilarity index was used to generate community distance matrices and used further in all community structure analysis. To address the relative importance of seasons and climatic zones on fungal community structure, multivariate permutational analysis of variance (PERMANOVA) was used, as implemented in the Adonis function of the package *vegan*. PERMANOVA analysis was executed using a forward selection practice to improve the final model^56^. First, we examined single variable models and thereafter, including significant factors in the final model in order of their R^2^ values. Furthermore, Nonmetric Multidimensional Scaling (NMDS) ordinations analyses were used to visualize the effects of studied factor variables on fungal community composition using the metaMDS function of the package *vegan*^57^. Centroids of the factors were fitted into NMDS plots using the function envfit. Ordiellipse function were used to plot the 95% confidence intervals (CI) of the different seasons.

To assess correspondence between overall mycotoxin and fungal community dataset, we used both mantel and procrustes analysis with 999 permutation, where distances matrixes of both datasets are compared together. In order to explore fungal community structural relationship between different mycotoxins in settled dust, airborne exposure levels of endotoxin, bacteria, fungal spores, and 1,3-beta glucans, blood concentrations of CC-16, SP-D, P-selectin, IL-6, TNFα, fibrinogen, CD40L and CRP, we further used NMDS ordination with the envfit function. In this test each explanatory variable is separately regressed on ordination axes 1 and 2 by linear regression analysis. The strength of the relationship between each variable and the ordination axes was assessed by the multiple coefficient of determination (R^2^) of this regression. A significance level of *p* < 0.05 was used throughout the study.

To further partition the variation of community dissimilarity from different seasons and climatic zone, we followed redundancy analysis (RDA) from the R package *vegan* (varpart function). The R^2^ values were adjusted according to each predictor (factor) level.

### Ethic statement

The Regional Ethical Committee of South-East Norway and the Norwegian Data Inspectorate approved the study. All participants gave their written informed consent upon participation in the study. All but one of the workers that received the written information agreed to participate with blood samples. The one that refused blood sampling gave written informed consent to participate in the exposure measurements only. All experiments were performed in accordance with relevant guidelines and regulations. The study was approved by the Regional Ethical Committee of South-East Norway, and received support from the Confederation of Norwegian Enterprise (S-2585).

## Supplementary information

Supplementary Informations.
