# Supplementary material for: The airborne mycobiome and associations with mycotoxins and inflammatory markers in the Norwegian grain industry
Source: Sci Rep. 2021 Apr 30;11:9357. doi: 10.1038/s41598-021-88252-1 (PMC8087811; doi:10.1038/s41598-021-88252-1)
Supplement: Supplementary file 1 — Supplementary Informations. [file 41598_2021_88252_MOESM1_ESM.docx]

**Supplementary material for**

The airborne mycobiome and associations with mycotoxins and inflammatory markers in the Norwegian grain industry

*Anne Straumfors^1#^, Sunil Mundra^,3^, Oda A.H. Foss^1^, Steen K. Mollerup^1^,and Håvard Kauserud^2^*

^1^Department of Chemical and Biological Work Environment, National Institute of Occupational Health, P.O. box 5330, Majorstuen, 0304 Oslo, Norway

^2^Department of Biosciences, Faculty of Mathematics and Natural Sciences, University of Oslo, Norway

^3^Department of Biology, College of Science, United Arab Emirates University (UAEU), P.O. Box. No. 15551, Al ain, Abu Dhabi, UAE

^#^Corresponding author: anne.straumfors@stami.no

**Fig. S1** Heat Tree illustrates hierarchical organization of overall fungal composition in Norwegian industrial grain mill air.


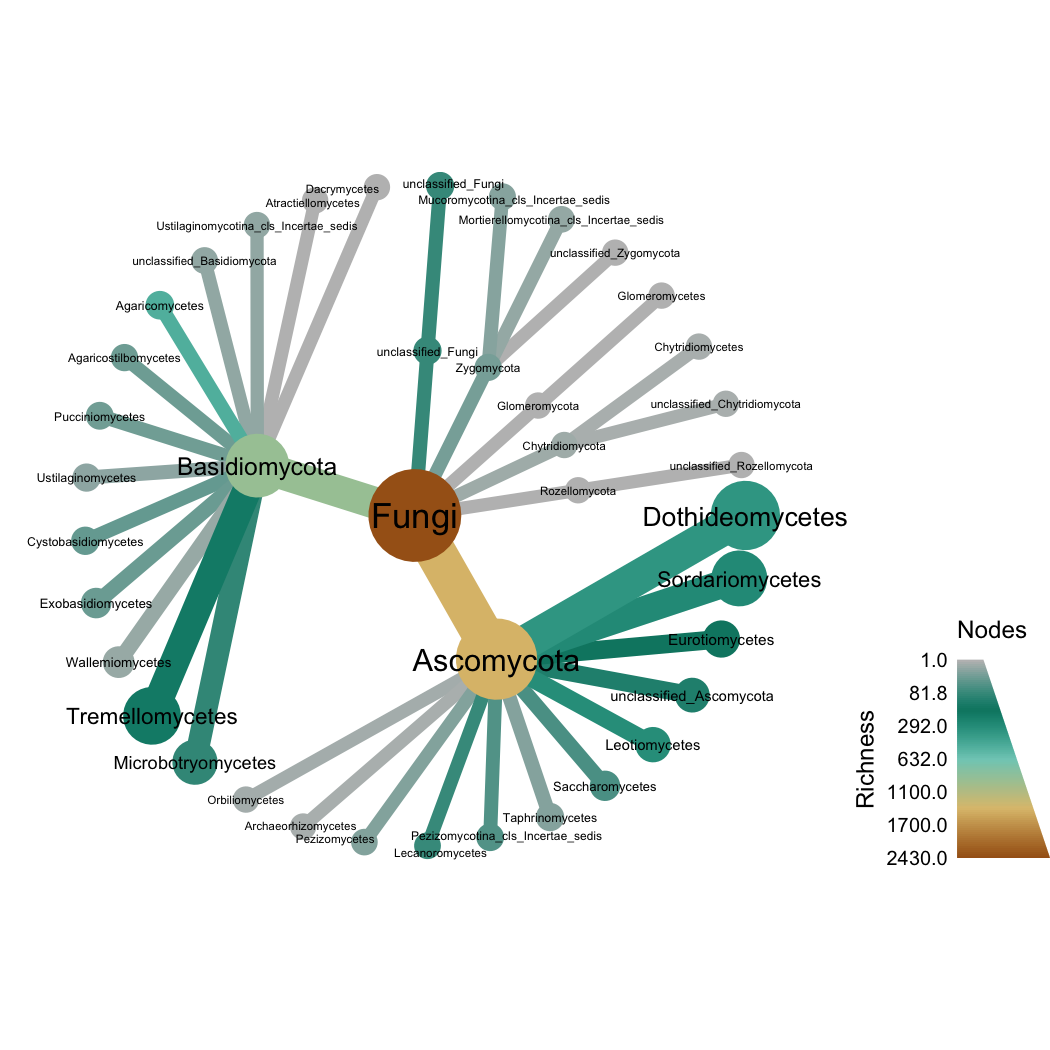


**Fig. S2** Violin plots showing pattern of fungal (a) richness, (b) evenness, (c) Shannon diversity index and (d) abundance of the 10 most common OTUs between two different seasons (autumn and winter). Statistically significant differences between seasons were analysed using ANOVA and Tukey’s HSD post-hoc test.

**
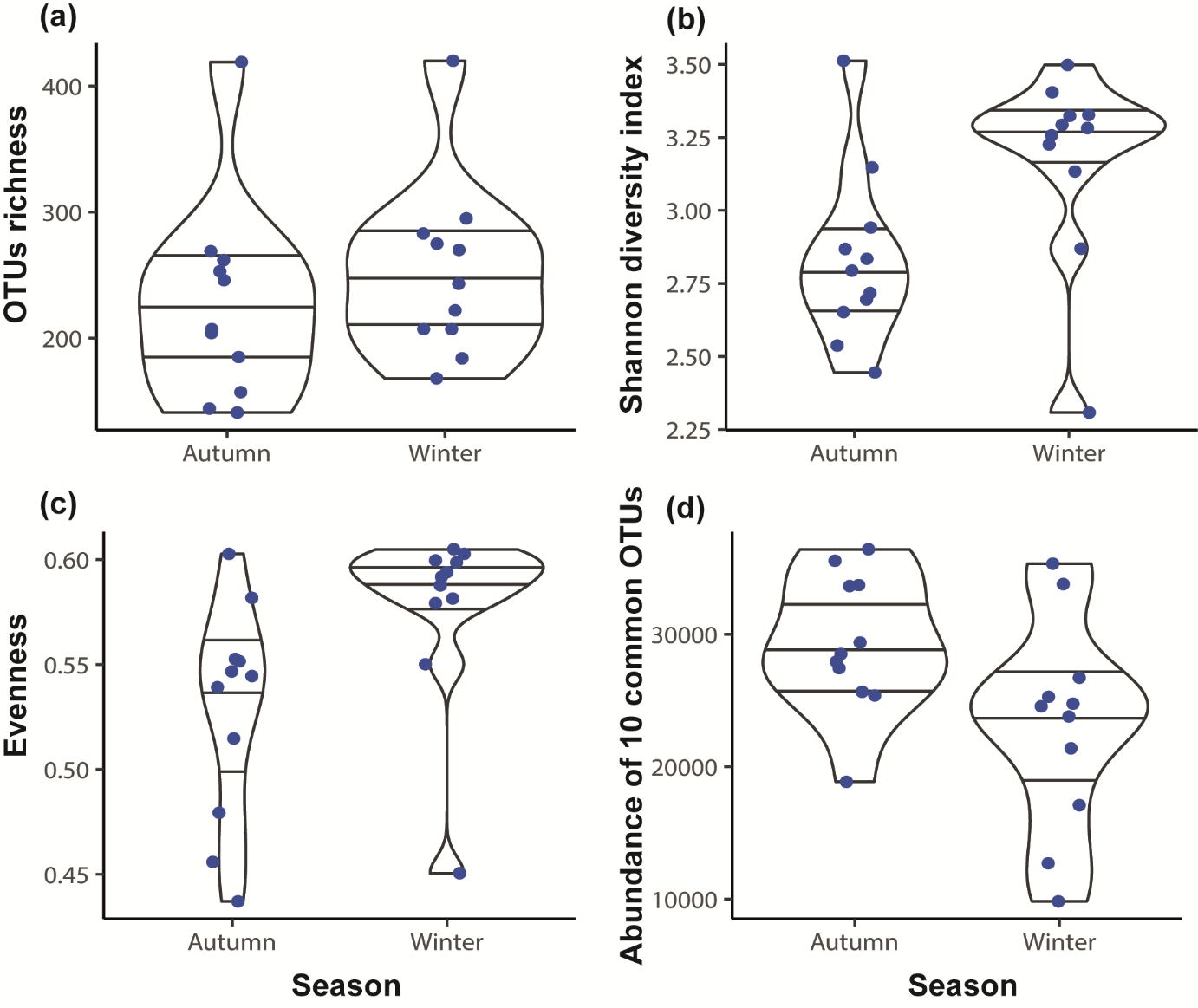
**

**Fig. S3** Violin plots showing pattern of fungal (a) richness, (b) evenness, (c) Shannon diversity index and (d) abundance of the 10 most common OTUs among different climatic zones. Statistically significant differences among climatic zones was analysed using ANOVA and Tukey’s HSD post-hoc test.

**
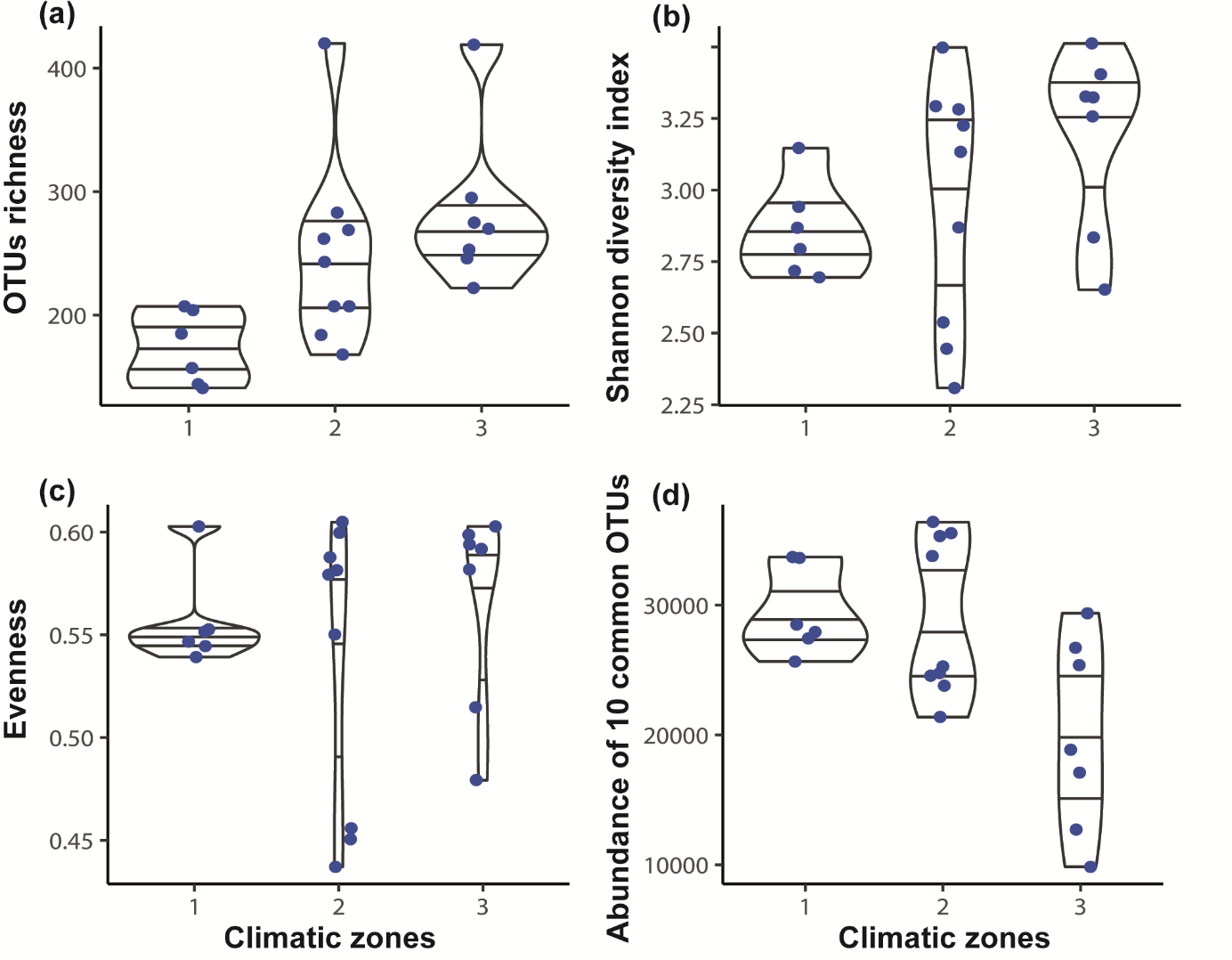
**

**Fig. S4** Pure and shared effects of different seasons (autumn and winter) and three climatic zones on the airborne fungal community in industrial grain mills as derived from variation partitioning analysis. Statistics denote the proportion of variation explained (adjusted R^2^) and values <0 are not shown.


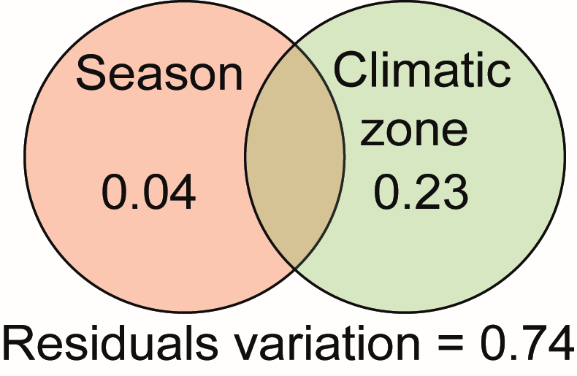


**Fig. S5** (a) Bar plots showing distribution of fungal reads per sample. (b) Rank-abundance plot displaying the number of reads per operational taxonomic units (OTUs).


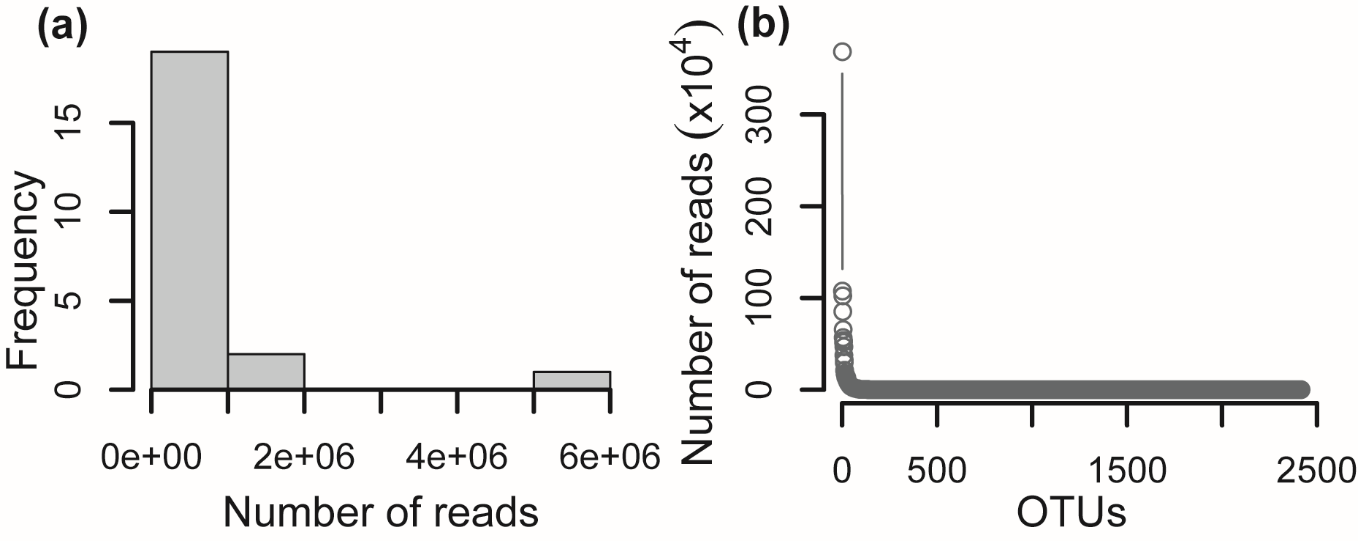


**Fig. S6** Rarefaction curves describing number of observed fungal operational taxonomic units (OTUs) as a function of number of sequencing reads. All diversity analyses were performed on rarefied dataset (43,837 reads per samples).

**
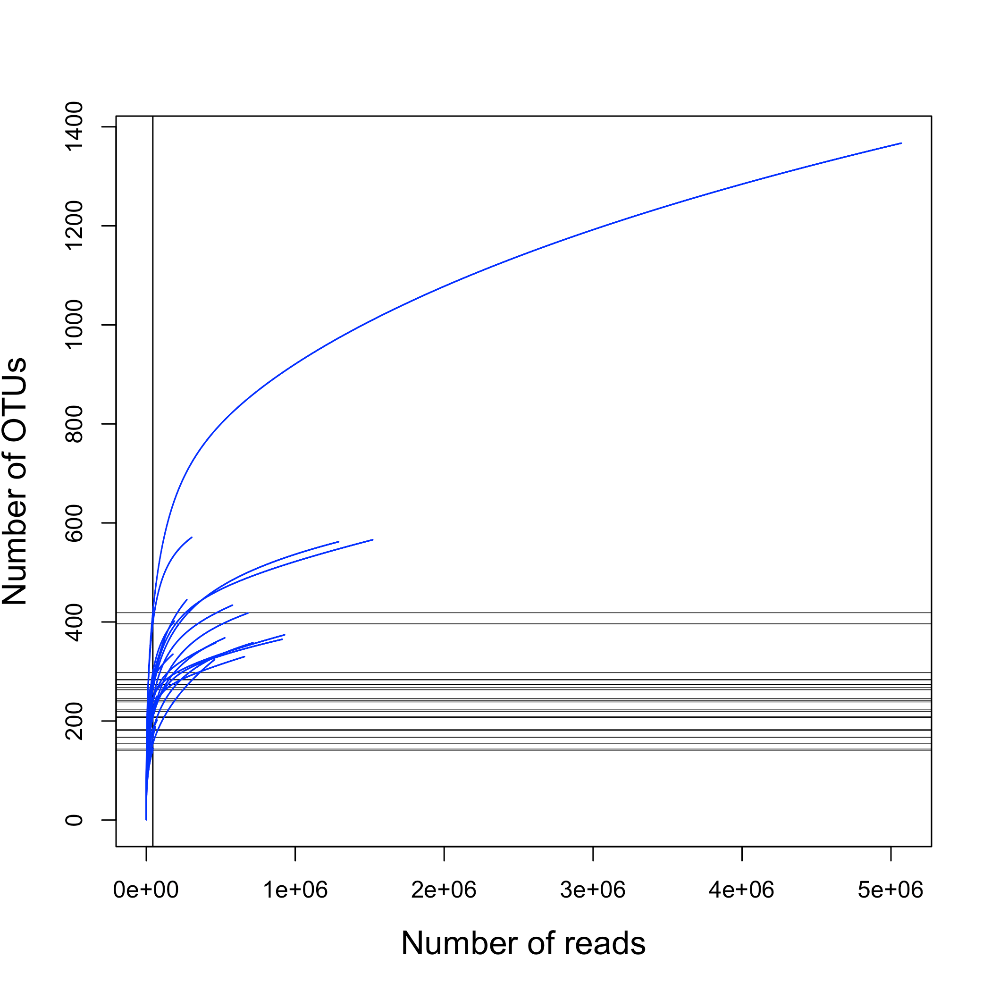
**

**Table S1** Taxonomic distribution of the grain mill fungal composition in terms of abundances (% reads) and occurrences (% OTUs) during different seasons (summer and winter) and three climatic zones.

| Taxonomy | Overall (%) | | Autumn (%) | | Winter (%) | | Climatic zone 1 (%) | | Climatic zone 2 (%) | | Climatic zone 3 (%) | |
| --- | --- | --- | --- | --- | --- | --- | --- | --- | --- | --- | --- | --- |
|  | Reads | OTUs | Reads | OTUs | Reads | OTUs | Reads | OTUs | Reads | OTUs | Reads | OTUs |
| Ascomycota | 68.2 | 59.2 | 69.2 | 55.1 | 67.4 | 62.7 | 37.7 | 51.0 | 71.6 | 62.1 | 81.1 | 59.4 |
| Capnodiales | 31.7 | 3.2 | 34.6 | 2.8 | 29.4 | 3.6 | 19.8 | 2.9 | 32.8 | 3.7 | 37.9 | 2.7 |
| Pleosporales | 9.2 | 10.1 | 6.9 | 9.6 | 11.1 | 10.6 | 2.6 | 8.0 | 8.4 | 10.0 | 18.1 | 11.5 |
| Hypocreales | 8.4 | 6.6 | 6.8 | 6.0 | 9.6 | 7.1 | 1.8 | 5.1 | 9.4 | 6.6 | 10.0 | 7.4 |
| Xylariales | 6.5 | 1.5 | 6.6 | 1.5 | 6.4 | 1.5 | 5.7 | 1.5 | 6.5 | 1.4 | 7.5 | 1.6 |
| Microascales | 4.6 | 1.1 | 8.3 | 1.1 | 1.6 | 1.1 | 0.0 | 1.0 | 6.6 | 1.1 | 0.5 | 1.0 |
| Eurotiales | 2.6 | 1.3 | 3.0 | 1.4 | 2.3 | 1.2 | 6.1 | 1.6 | 1.9 | 1.2 | 2.4 | 1.2 |
| Helotiales | 1.8 | 8.7 | 0.4 | 8.3 | 2.9 | 9.1 | 0.2 | 8.3 | 2.4 | 9.1 | 0.6 | 8.3 |
| Un Ascomycota | 1.6 | 4.7 | 1.4 | 4.4 | 1.8 | 5.0 | 0.4 | 3.7 | 1.7 | 5.2 | 2.4 | 4.5 |
| Dothideales | 0.5 | 0.6 | 0.3 | 0.6 | 0.8 | 0.6 | 0.4 | 0.6 | 0.6 | 0.6 | 0.4 | 0.6 |
| Saccharomycetales | 0.4 | 2.0 | 0.4 | 2.0 | 0.4 | 2.1 | 0.2 | 2.2 | 0.4 | 1.9 | 0.4 | 2.1 |
| Un Dothideomycetes | 0.2 | 1.9 | 0.1 | 1.8 | 0.2 | 2.0 | 0.0 | 1.4 | 0.2 | 2.0 | 0.4 | 2.2 |
| Entylomatales | 0.2 | 0.4 | 0.1 | 0.5 | 0.2 | 0.4 | 0.0 | 0.6 | 0.2 | 0.3 | 0.1 | 0.4 |
| Dothideomycetes IS | 0.1 | 1.4 | 0.1 | 1.4 | 0.2 | 1.4 | 0.0 | 1.3 | 0.1 | 1.4 | 0.2 | 1.5 |
| Basidiomycota | 31.7 | 37.1 | 30.7 | 41.3 | 32.5 | 33.4 | 62.2 | 45.5 | 28.3 | 34.1 | 18.8 | 36.9 |
| Tremellales | 17.9 | 8.6 | 17.2 | 9.1 | 18.5 | 8.1 | 34.5 | 11.5 | 16.1 | 8.2 | 10.8 | 7.6 |
| Sporidiobolales | 7.8 | 1.5 | 8.2 | 1.4 | 7.4 | 1.6 | 16.7 | 1.4 | 6.8 | 1.5 | 3.8 | 1.5 |
| Cystofilobasidiales | 3.2 | 2.1 | 3.5 | 2.6 | 3.0 | 1.7 | 8.8 | 3.3 | 2.5 | 1.8 | 1.2 | 1.9 |
| Filobasidiales | 1.0 | 0.7 | 0.5 | 0.7 | 1.4 | 0.6 | 0.7 | 0.8 | 1.0 | 0.6 | 0.9 | 0.8 |
| Wallemiales | 0.7 | 0.5 | 0.4 | 0.5 | 0.9 | 0.5 | 0.6 | 0.5 | 0.7 | 0.5 | 0.4 | 0.6 |
| Un Exobasidiomycetes | 0.2 | 0.4 | 0.1 | 0.4 | 0.3 | 0.4 | 0.1 | 0.5 | 0.3 | 0.3 | 0.2 | 0.5 |
| Un Tremellomycetes | 0.2 | 0.9 | 0.1 | 0.9 | 0.2 | 0.8 | 0.3 | 1.0 | 0.1 | 0.8 | 0.1 | 1.0 |
| Microbotryales | 0.1 | 1.0 | 0.1 | 0.9 | 0.1 | 1.1 | 0.3 | 1.0 | 0.1 | 0.9 | 0.1 | 1.2 |
| unclassified_Fungi | 0.1 | 2.8 | 0.1 | 2.6 | 0.1 | 2.9 | 0.0 | 2.6 | 0.1 | 2.9 | 0.2 | 2.7 |
| Zygomycota | 0.0 | 0.8 | 0.0 | 0.8 | 0.0 | 0.8 | 0.0 | 0.9 | 0.0 | 0.8 | 0.0 | 0.9 |
| Glomeromycota | 0.0 | 0.0 | 0.0 | 0.0 | 0.0 | 0.0 | 0.0 | 0.0 | 0.0 | 0.0 | 0.0 | 0.0 |
| Chytridiomycota | 0.0 | 0.1 | 0.0 | 0.0 | 0.0 | 0.1 | 0.0 | 0.1 | 0.0 | 0.1 | 0.0 | 0.1 |
| Rozellomycota | 0.0 | 0.0 | 0.0 | 0.0 | 0.0 | 0.0 | 0.0 | 0.0 | 0.0 | 0.0 | 0.0 | 0.0 |

Abbreviations: Un = unidentified; IS = Incertae sedis

**Table S2**. Mycotoxins and fungal metabolites in settled grain dust (µg/kg dust):

| **Fungal metabolite** | **AM** | **Min** | **Max** |
| --- | --- | --- | --- |
| T2-tetraol | 6.2 | 0.0 | 31 |
| T-2 toxin | 39.4 | 0.0 | 127 |
| HT-2 toxin | 147 | 0.0 | 730 |
| Neosolaniol | 0.8 | 0.0 | 15 |
| Nivalenol | 37.3 | 5.1 | 84 |
| Deoxynivalenol | 1919 | 122 | 6689 |
| DON-3-glycoside | 78.3 | 12.2 | 253 |
| 3-Acetyl-deoxynivalenol | 58.1 | 0.0 | 317 |
| Chanoclavine | 1.9 | 0.0 | 15 |
| Agroclavine | 0.3 | 0.0 | 6 |
| Fumigaclavine | 0.1 | 0.0 | 1 |
| Ergometrine | 7.9 | 0.0 | 55 |
| Ergometrinine | 3.6 | 0.0 | 27 |
| Ergocristine | 2.4 | 0.0 | 23 |
| Ergocristinine | 1.5 | 0.0 | 11 |
| Ergosine | 1.8 | 0.0 | 11 |
| Chlamydosporols | 42.4 | 0.0 | 186 |
| Aurofusarin | 8678 | 494 | 34968 |
| Avenacein Y | 3637 | 673 | 8444 |
| Moniliformin | 274 | 57.2 | 1089 |
| Butenolide | 199 | 0.0 | 1650 |
| Enniatin A | 118 | 3.7 | 675 |
| Enniatin A1 | 773 | 26.5 | 4110 |
| Enniatin B | 869 | 98.8 | 2055 |
| Enniatin B1 | 1510 | 87.7 | 6000 |
| Enniatin B2 | 68.7 | 6.2 | 238 |
| Enniatin B3 | 0.3 | 0.1 | 1 |
| Beauvericin | 45.5 | 3.4 | 204 |
| Fumonisin B1 | 257 | 0.0 | 993 |
| Fumonisin B2 | 104 | 0.0 | 405 |
| Fumonisin B3 | 16.9 | 0.0 | 72 |
| Culmorin | 2738 | 231 | 9312 |
| 15-OH-Culmorin | 455 | 0.0 | 1873 |
| Zearalenone | 101 | 4.4 | 324 |
| b-Zearalenol | 4.9 | 0.0 | 27 |
| Zearalenone-4-sulphate | 8.9 | 0.0 | 39 |
| Alternariol | 95.2 | 0.0 | 312 |
| Alternariol-OMe | 8.9 | 2.2 | 29 |
| Altertoxin-I | 18.3 | 0.0 | 67 |
| Tentoxin | 4.1 | 0.0 | 16 |
| **Fungal metabolite** | **AM** | **Min** | **Max** |
| Equisetin | 287 | 32.2 | 943 |
| Secalonic acid | 46.6 | 0.0 | 235 |
| Sterigmatocystin | 2.7 | 0.0 | 11 |
| Mycophenolic acid | 57.9 | 10.9 | 135 |
| Ochratoxin A | 3.1 | 0.0 | 15 |
| Dechlorogriseofulvin | 47.6 | 0.0 | 242 |
| Averufin | 0.0 | 0.0 | 0 |
| Curvularin | 33.0 | 0.0 | 159 |
| Asterric acid | 9.2 | 0.0 | 37 |
| 3-Nitropropionic acid | 16.0 | 0.0 | 47 |
| Emodin | 97.5 | 3.2 | 298 |
| Cyclopenol | 375 | 0.0 | 3732 |
| Cyclopenine | 11.6 | 0.0 | 143 |
| Nonactin | 3.9 | 0.0 | 32 |
| Monactin | 8.3 | 0.0 | 57 |
| Monocerin | 5.3 | 2.2 | 14 |
| Tryptophol | 59.5 | 0.0 | 137 |
| Viridicatin | 5.4 | 0.0 | 36 |
| 3-OMe-viridicatin | 5.7 | 0.0 | 24 |
| Viomellein | 184 | 0.0 | 1726 |
| Terphenyllin | 25.4 | 0.0 | 232 |
| Cyclopeptine | 2.5 | 0.0 | 16 |
| Brevianamid F | 28.8 | 3.0 | 140 |
| Meleagrin | 9.1 | 0.0 | 57 |
| Rubellin D | 19.2 | 0.0 | 157 |
| Apicidin | 73.7 | 4.1 | 381 |
| Secalonic acid D | 46.6 | 0.0 | 235 |
| Curvularin | 33.0 | 0.0 | 159 |
| Cyclosporin C | 114 | 0.0 | 1030 |
| Calphostin C | 73.1 | 0.0 | 452 |
| Methylsulochrin | 1.6 | 0.0 | 6 |
| Chrysophanol | 22.9 | 0.0 | 77 |
| Skyrin | 15.7 | 0.0 | 51 |
| Physcion | 545 | 0.0 | 4531 |

Number of samples n=19; the co-occurrence of multiple mycotoxins and other fungal metabolites in grain dust has been published previously (Straumfors *et al*. 2014)

**Table S3**. Personal bioaerosol exposure levels among grain industry workers

| Company number | Climatic zone | Season | Grain dust (mg/m^3^) | Total fungal spores (#×10^4^/m^3^) | 1,3-β-glucan (µg/m^3^) |
| --- | --- | --- | --- | --- | --- |
| 1 | 2 | Winter | 5.16 | 216 | 73.91 |
| 2 | 2 | Winter | 12.44 | 7.4 | 146.58 |
| 3 | 2 | Winter | 0.41 | 1.7 | 9.07 |
| 4 | 2 | Winter | 1.45 | 2.5 | 4.95 |
| 5 | 2 | Winter | 3.13 | 3.8 | 24.81 |
| 6 | 3 | Winter | 4.55 | 67.6 | 45.12 |
| 7 | 3 | Winter | 2.58 | 2.7 | 19.19 |
| 8 | 3 | Winter | 1.20 | 1.3 | 10.66 |
| 9 | 3 | Winter | 0.98 | 4.2 | 19.67 |
| 10 | 1 | Autumn | 1.70 | 11.1 | 52.26 |
| 11 | 1 | Autumn | 0.38 | 2.6 | 7.83 |
| 12 | 1 | Autumn | 0.38 | 2.2 | 3.78 |
| 13 | 1 | Autumn | 1.48 | 5.7 | 12.72 |
| 14 | 1 | Autumn | 1.14 | 12.3 | 9.86 |
| 15 | 1 | Autumn | 0.84 | 1.9 | 3.39 |
| 16 | 2 | Autumn | 1.20 | 13.0 | 41.75 |
| 17 | 2 | Autumn | 3.00 | 18.3 | 20.09 |
| 18 | 3 | Autumn | 4.37 | 10.1 | 124.25 |
| 19 | 3 | Autumn | 0.41 | 1.6 | 2.09 |
| 20 | 3 | Autumn | 0.80 | 12.8 | 16.89 |
| Mean exposure: | | | 2.4 | 20 | 32.4 |

The exposure levels are mean of all personal measurements (n=130) by company (n=20). Grain dust were analyzed by gravimetric assessment of mass, total fungal spores by scanning electron microscopy and 1,3-β-glucan by EIA. Personal sampling, the analytical methods, and individual exposure levels are described previously (Halstensen *et al.* 2013)

**Table S4.** Biomarkers in the blood of grain industry workers

| Company number | Climatic zone | Season | CC-16 (ng/ml) | SPD (ng/ml) | SPA (µg/ml) | sP-selektin (ng/ml) | IL-6 (pg/ml) | TNF-α (pg/ml) | Fibrinogen (mg/ml) | sCD40L (ng/ml) | mCRP (mg/L) |
| --- | --- | --- | --- | --- | --- | --- | --- | --- | --- | --- | --- |
| 1 | 2 | Winter | 3.33 | 57.90 | 178.73 | 53.18 | 1.37 | 0.85 | 2.81 | 3.89 | 1.60 |
| 2 | 2 | Winter | 6.19 | 126.38 | 109.15 | 63.57 | 4.27 | 1.02 | 3.85 | 3.56 | 3.36 |
| 3 | 2 | Winter | 6.03 | 68.65 | 541.05 | 61.42 | 1.61 | 0.75 | 3.67 | 8.17 | 0.65 |
| 4 | 2 | Winter | 6.08 | 163.87 | 172.17 | 73.68 | 3.37 | 1.42 | 4.23 | 1.61 | 5.80 |
| 5 | 2 | Winter | 5.29 | 103.27 | 27.64 | 57.06 | 1.63 | 0.96 | 3.22 | 2.90 | 1.35 |
| 6 | 3 | Winter | 6.99 | 135.80 | 68.55 | 37.87 | 1.79 | 1.07 | 3.39 | 2.15 | 3.67 |
| 7 | 3 | Winter | 4.60 | 153.20 | 60.29 | 55.80 | 2.52 | 1.43 | 3.22 | 2.56 | 1.75 |
| 8 | 3 | Winter | 6.68 | 109.17 | 39.26 | 49.66 | 1.23 | 0.85 | 3.59 | 1.26 | 1.87 |
| 9 | 3 | Winter | 4.69 | 99.42 | 82.24 | 67.65 | 1.52 | 1.15 | 2.85 | 1.98 | 1.06 |
| 10 | 1 | Autumn | 5.24 | 155.50 | 339.18 | 64.28 | 1.02 | 1.26 | 3.07 | 4.84 | 0.75 |
| 11 | 1 | Autumn | 6.42 | 194.67 | 107.66 | 55.13 | 1.86 | 0.93 | 3.09 | 3.00 | 3.00 |
| 12 | 1 | Autumn | 6.20 | 80.90 | 170.46 | 45.50 | 0.84 | 1.09 | 2.49 | 4.30 | 2.45 |
| 13 | 1 | Autumn | 4.18 | 89.17 | 80.67 | 72.81 | 2.43 | 1.10 | 4.16 | 0.94 | 2.73 |
| 14 | 1 | Autumn | 3.41 | 126.53 | 53.92 | 60.80 | 1.87 | 1.11 | 2.41 | 3.48 | 3.50 |
| 15 | 1 | Autumn | 6.08 | 100.28 | 261.30 | 44.54 | 3.65 | 1.07 | 3.03 | 1.73 | 4.15 |
| 16 | 2 | Autumn | 4.08 | 114.53 | 47.27 | 53.59 | 1.83 | 1.27 | 3.24 | 0.81 | 1.70 |
| 17 | 2 | Autumn | 7.72 | 164.53 | 223.37 | 41.66 | 4.33 | 1.24 | 3.22 | 3.38 | 1.17 |
| 18 | 3 | Autumn | 3.86 | 92.38 | 74.05 | 37.80 | 2.91 | 1.20 | 3.89 | 1.20 | 3.98 |
| 19 | 3 | Autumn | 4.08 | 116.85 | 419.68 | 37.44 | 3.42 | 1.37 | 3.59 | 3.95 | 5.18 |
| 20 | 3 | Autumn | 5.19 | 139.25 | 109.52 | 60.86 | 3.68 | 1.86 | 3.83 | 1.92 | 11.40 |
| Mean blood levels of exposed | | | 5.3 | 119.6 | 158.3 | 54.7 | 2.4 | 1.1 | 3.3 | 2.9 | 3.1 |
| Mean blood levels of controls | | | 3.9 | 113.3 | 120.3 | 58.8 | 1.5 | 1.1 | 4.1 | 2.9 | 2.5 |

aThe mean concentrations of biomarkers in blood of exposed workers by company. The blood levels are mean of all individual measurements of exposed workers (n=67) by company (n=20). The mean of all exposed and all unexposed controls (n=37) are given at the bottom of the table for comparison. The differences of biomarker levels in blood between exposed workers and controls and the relationship with exposure and lung function are described previously (Straumfors *et al.* 2018).

**Table S5.** Significance of vectors (mycotoxins and other variables) that were fitted to the NMDS ordination of the airborne fungal communities in Norwegian industrial grain mills. Variables with significant relationship (p < 0.05) with NMDS ordination are shown in bold face.

| Variable | R2 | p value |
| --- | --- | --- |
| T2-tetraol (ug/kg dust) | 0.02 | 0.834 |
| T-2 toxin | 0.22 | 0.144 |
| HT-2 toxin | 0.11 | 0.403 |
| Nivalenol | 0.01 | 0.925 |
| Deoxynivalenol | 0.07 | 0.537 |
| DON-3-glycoside | 0.01 | 0.922 |
| 3-Acetyl-deoxynivalenol | 0.01 | 0.948 |
| Chanoclavine | 0.11 | 0.435 |
| Ergometrine | 0.09 | 0.473 |
| Ergosine | 0.05 | 0.687 |
| Chlamydosporols | 0.16 | 0.275 |
| Aurofusarin | 0.07 | 0.552 |
| Avenacein Y | 0.30 | 0.076 |
| Moniliformin | 0.17 | 0.242 |
| Butenolide | 0.18 | 0.207 |
| Enniatin A | 0.35 | 0.054 |
| Enniatin A1 | **0.42** | **0.030** |
| Enniatin B | **0.38** | **0.037** |
| Enniatin B1 | **0.40** | **0.038** |
| Enniatin B2 | 0.32 | 0.072 |
| Enniatin B3 | **0.40** | **0.029** |
| Beauvericin | 0.07 | 0.567 |
| Fumonisin B1 | 0.07 | 0.584 |
| Fumonisin B2 | 0.06 | 0.637 |
| Culmorin | 0.04 | 0.721 |
| 15-OH-Culmorin | 0.07 | 0.572 |
| Zearalenone | 0.13 | 0.350 |
| b-Zearalenol | 0.01 | 0.932 |
| Zearalenone-4-sulphate | 0.15 | 0.295 |
| Alternariol | 0.33 | 0.058 |
| Alternariol-OMe | 0.11 | 0.389 |
| Altertoxin-I | 0.22 | 0.162 |
| Tentoxin | 0.05 | 0.648 |
| Equisetin | 0.28 | 0.094 |
| Sterigmatocystin | 0.09 | 0.490 |
| Mycophenolic acid | 0.28 | 0.097 |
| Dechlorogriseofulvin | 0.00 | 0.982 |
| Curvularin | 0.28 | 0.079 |
| Asterric acid | **0.43** | **0.016** |
| 3-Nitropropionic acid | 0.27 | 0.091 |
| Emodin | 0.28 | 0.100 |
| Cyclopenol | 0.12 | 0.372 |
| Cyclopenine | 0.07 | 0.596 |
| Nonactin | 0.00 | 0.992 |
| Monactin | 0.04 | 0.700 |
| Monocerin | 0.12 | 0.373 |
| Tryptophol | 0.11 | 0.447 |
| Viridicatin | 0.01 | 0.915 |
| 3-OMe-viridicatin | 0.02 | 0.847 |
| Viomellein | 0.18 | 0.220 |
| Cyclopeptine | 0.06 | 0.627 |
| Brevianamid F | 0.04 | 0.691 |
| Rubellin D | **0.42** | **0.011** |
| Apicidin | 0.17 | 0.269 |
| Curvularin | 0.28 | 0.079 |
| Calphostin C | 0.16 | 0.254 |
| Methylsulochrin | 0.02 | 0.866 |
| Chrysophanol | 0.04 | 0.736 |
| Skyrin | 0.17 | 0.221 |
| Physcion | 0.13 | 0.350 |
| ΣMycotoxins and metabolites | 0.16 | 0.298 |
| Dust (mg/m^3^) | 0.16 | 0.250 |
| Fungal spores (#/m^3^) | 0.02 | 0.850 |
| 1,3-beta-glucan (µg/m^3^) | 0.07 | 0.593 |
| CC-16 (ng/ml) | 0.05 | 0.662 |
| SPD (ng/ml) | 0.01 | 0.941 |
| SPA(µg/ml) | 0.11 | 0.412 |
| sP-selectin (ng/ml) | 0.14 | 0.320 |
| IL-6 (pg/ml) | 0.01 | 0.898 |
| TNF-α (pg/ml) | 0.05 | 0.692 |
| Fibrinogen (mg/ml) | **0.35** | **0.045** |
| sCD40L (ng/ml) | 0.13 | 0.355 |
| CRP | 0.04 | 0.763 |
